# Supplementary material for: Chemokines form complex signals during inflammation and disease that can be decoded by extracellular matrix proteoglycans
Source: Sci Signal. Author manuscript; Available in PMC 2025 Jul 20. (PMC7617913; doi:10.1126/scisignal.adf2537)
Supplement: Supplementary materials [file EMS207236-supplement-Supplementary_materials.docx]

**Supplementary Figure 1. Schematic explanation of data analysis from the EMBL-ELI expression atlas.**

**Supplementary Figure 2. Human tissues have tissue specific chemokine ligand and receptor expression patterns.** EMBL-ELI expression atlas heat map analysis of chemokine ligands and receptors across human brain, lungs and lymph node.

**Supplementary Figure 3. Mouse tissues have tissue specific chemokine ligand and receptor expression patterns.** EMBL-ELI expression atlas PCA and heat map analysis of chemokine ligands and receptors across mouse pooled tissues, mouse brain, lungs and lymph node.

**Supplementary Figure 4. Chemokine receptors have different transcriptional relationships with their ligands.** EMBL-ELI expression atlas heat map analysis of Pearson correlation coefficient between CXCR1, 2, 3, 4, 5 and 5 and their individual ligands.

**Supplementary Figure 5. Chemokine receptors have different transcriptional relationships with their ligands.** EMBL-ELI expression atlas heat map analysis of Pearsons correlation between CCR1, 2, 3 and 5 and their individual ligands.

**Supplementary Figure 6. Chemokine receptors have different transcriptional relationships with their ligands.** EMBL-ELI expression atlas heat map analysis of Pearsons correlation between CCR4, 6, 7, 8, 9 and 10 and their individual ligands.

**Supplementary Figure 7. Flow cytometric gating strategy for identifying air pouch fluid derived immune cells.** Cells isolated from air pouch fluid were stained and cell populations (highlighted in green) were defined based on their expression profiles of the surface markers stated. Plots are representative of >3 experiments although the proportions of different cell types differed. Numbers adjacent to boxed areas indicate the percentages of cells that fall within each gate.

**Supplementary Figure 8. tSNE analysis of cell populations flushed from the air pouch after chemokine injection.** Representative tSNE analysis of cells flushed from the chemokine stimulated air pouch for expression of the indicated flow cytometry markers.

**Supplementary Figure 9. CXCR3 ligands do not recruit neutrophils, macrophages or eosinophils to the air pouch.** CXCL9, 10 and 11 were injected into the air pouch and 24 hrs later cells were analysed to quantify the number of recruited neutrophils, macrophages or eosinophils. Data plotted ± SEM from three pooled separate experiments where each dot represents an individual mouse, analysed using a one-way ANOVA with a Sidak multiple comparison test.

**Supplementary Figure 10. ECM proteoglycan GAG synthesis, sulphation and protein core genes show little transcriptional correlation.** EMBL-ELI expression atlas heat map analysis of Pearsons correlation between genes that mediate (A) GAG chains synthesis, (B) GAG chain sulphation or (C) proteoglycan protein core production.

**Supplementary Figure 11. Human ECM GAG genes have tissue specific correlation signatures.** EMBL-ELI expression atlas PCA and heat map analysis of Pearsons correlation between genes involved in ECM GAG synthesis and modification across human tissues.

**Supplementary Figure 12. Mouse ECM GAG genes have tissue specific correlation signatures.** EMBL-ELI expression atlas PCA and heat map analysis of Pearsons correlation between genes involved in ECM GAG synthesis and modification across murine tissues.

**Supplementary Figure 13. Analysis of CXCL10 and CXCL11 mediated T cell recruitment to the air pouch with and without heparin. (A)** CXCL10 or (B) CXCL11 were injected into the air pouch and 24 hrs later cells were analysed to quantify the number of recruited T cell subsets with or without pre-incubation with heparin. Data plotted ± SEM from three pooled separate experiments where each dot represents an individual mouse, analysed using a one-way ANOVA with a Sidak multiple comparison test.


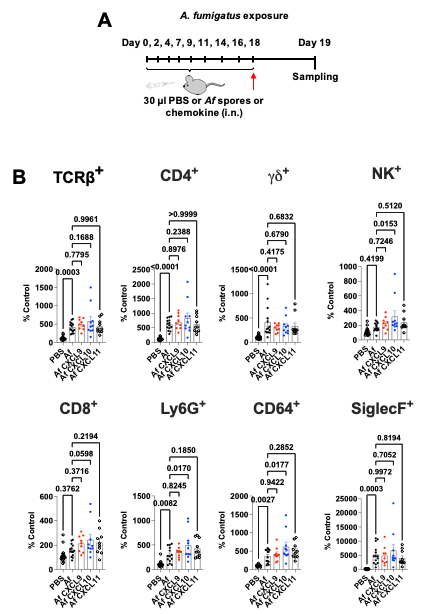


**Supplementary Figure 14. Analysis of CXCL9, CXCL10 and CXCL11 mediated T cell recruitment to the lungs of mice exposed to *A. fumigatus*. (A)** Schematic of the *A. fumigatus* exposure model. (B) CXCL9, CXCL10 or CXCL11 were administered intranasally and 24 hrs later lungs were analysed to quantify the number of recruited cell subsets with. Data plotted ± SEM from two pooled separate experiments where each dot represents an individual mouse, analysed using a one-way ANOVA with a Sidak multiple comparison test.
